# Comparative Cytogenetics between Two Important Songbird, Models: The Zebra Finch and the Canary

**DOI:** 10.1371/journal.pone.0170997

**Published:** 2017-01-27

**Authors:** Michelly da Silva dos Santos, Rafael Kretschmer, Carolina Frankl-Vilches, Antje Bakker, Manfred Gahr, Patricia C. M. O´Brien, Malcolm A. Ferguson-Smith, Edivaldo H. C. de Oliveira

**Affiliations:** 1 Programa de Pós-Graduação em Genética e Biologia Molecular, ICB, UFPA, Belém, PA, Brazil; 2 Programa de Pós-Graduação em Genética e Biologia Molecular, UFRGS, Porto Alegre, RS, Brazil; 3 Department of Behavioral Neurobiology, Max Planck Institute for Ornithology, Seewiesen, Germany; 4 Cambridge Resource Centre for Comparative Genomics, University of Cambridge Department of Veterinary Medicine, Cambridge, United Kingdom; 5 Laboratório de Cultura de Tecidos e Citogenética, SAMAM, Instituto Evandro Chagas, Ananindeua, PA, Brazil; 6 Faculdade de Ciências Naturais, ICEN, Universidade Federal do Pará, Belém, Brazil; Utrecht University, NETHERLANDS

## Abstract

Songbird species (order Passeriformes, suborder Oscines) are important models in various experimental fields spanning behavioural genomics to neurobiology. Although the genomes of some songbird species were sequenced recently, the chromosomal organization of these species is mostly unknown. Here we focused on the two most studied songbird species in neuroscience, the zebra finch (*Taeniopygia guttata*) and the canary (*Serinus canaria*). In order to clarify these issues and also to integrate chromosome data with their assembled genomes, we used classical and molecular cytogenetics in both zebra finch and canary to define their chromosomal homology, localization of heterochromatic blocks and distribution of rDNA clusters. We confirmed the same diploid number (2n = 80) in both species, as previously reported. FISH experiments confirmed the occurrence of multiple paracentric and pericentric inversions previously found in other species of Passeriformes, providing a cytogenetic signature for this order, and corroborating data from *in silico* analyses. Additionally, compared to other Passeriformes, we detected differences in the zebra finch karyotype concerning the morphology of some chromosomes, in the distribution of 5S rDNA clusters, and an inversion in chromosome 1.

## Introduction

Species belonging to the suborder Oscines (Aves, order Passeriformes), also known as songbirds, have been employed as models in studies concerning neuroscience, vocal communication, development, behavioural genomics, ecology and evolution, among others [1–7]. Among songbirds, the zebra finch (*Taeniopygia guttata*, TGU) and the canary (*Serinus canaria*, SCA) belong to different families (Estrildidae and Fringillidae, respectively), are frequently used and have recently been the subjects of genomic analyses [8–11]. They are originally from different zoogeographical regions–the zebra finch is an Australian species, while the canary originates from the Canary Islands, located just off the southern coast of Africa [12].

After the chicken (*Gallus gallus*, GGA) the zebra finch was the second bird species to have its genome sequenced [8] which generated knowledge crucial in understanding some aspects of genome evolution in birds. Comparisons with data collected from sequencing projects of turkey (*Meleagris gallopavo*) and chicken revealed numerous intrachromosomal rearrangements, 10% of which are recurrent, indicating the existence of evolutionary hotspots [3]. The canary genome was sequenced and analyzed in a study dealing with the evolution of the influence of sex related hormones in gene regulation, in which an *in silico* karyotype was proposed, based on the alignment of the canary genome with 13 other birds, including the zebra-finch [11]. Interestingly, Romanov et al. [13] found that the zebra finch and budgerigar–representing lineages with vocal learning—showed the highest intrachromosomal rearrangement rates among birds. More recently, Farré et al. [14] published a study demonstrating how chromosomal rearrangements act as a source of phenotypic variation. Considering Passeriformes, the comparison between Oscines and Suboscines showed that the number of chromosomal rearrangements is higher in birds with vocal learning [6, 13].

In cytogenetic studies, *G*. *gallus* is also used as the main model for comparisons. Indeed, the comparison of data obtained by chromosome painting of different species of birds led Griffin et al. [15] to propose a putative avian ancestral karyotype (PAK), which showed a total correspondence to syntenic groups of *G*. *gallus*, except for the pair 4, which corresponds to two elements in the PAK (pairs 4 and 10), as found in most species of birds so far. In addition, the use of white hawk (*Leucopternis albicollis*, LAL) whole-chromosome paints brought new information concerning chromosomal rearrangements in birds, because many of its chromosomes correspond to regions of macrochromosomes of the PAK, and hence allow identification of the occurrence of chromosomal rearrangements, such as paracentric inversions, and breakpoints [16].

Most species of songbirds, including the zebra finch and the canary, show a diploid number close to 2n = 80. Although GGA whole chromosome probes have been used in the zebra-finch to detect some specific intrachromosomal polymorphisms little is known about the karyotype of the canary apart from the first description based on conventional staining [17]. So far, 14 species of songbirds have been analyzed by chromosome painting, most of them with GGA probes [9, 18–23]. These studies confirm the conservation of most syntenic groups found in the putative avian ancestral karyotype (PAK). An apparent centric fission in PAK chromosome 1 (GGA1) may represent a synapomorphy for the Oscines.

More recently, the use of two sets of whole chromosome probes (GGA and LAL) in the analysis of the karyotype of different species of Oscines and Suboscines from South America have confirmed the centric fission of PAK 1 as a synapomorphy, and revealed a complex pattern of paracentric and pericentric inversions in the pair corresponding to PAK 1q (GGA 1q) as a chromosome signature of this order. The results obtained corroborated the findings observed *in silico* [3], concerning the confirmation of the occurrence of inversions, and allowed determination of the possible sequence of some events, due to some differences among the inversions in each of the species [21, 23]. However, these studies have been performed only in South American species of Passeriformes, where the species of Oscines belonged to families Turdidae and Thraupidae.

Therefore, in order to verify and corroborate the occurrence of such intrachromosomal rearrangements in other groups of Passeriformes, and because of the importance of the zebra finch and the canary as biological models, we present here the chromosomal analysis of these two species by classical and molecular cytogenetics, using not only whole-chromosome probes of chicken and the white-hawk, but also 5S and 18S rDNA probes, and telomeric sequences.

## Material and Methods

### Cell culture and chromosome isolation

This study was approved by the Ethics Committee on the Use of Animals (CEUA-Universidade Federal do Pará, Permission Number: 070/2013). Tissue cultures were initiated from embryos from eggs after five to six days (HH stages 27–29, approximately) of incubation at 37°C. Sex determination was performed by molecular techniques, and cultures of one male and one female of zebra finch and canary were selected. Tissue was cut into small pieces and disaggregated in 0.5% collagenase IV for 30 min. The suspension was then washed with culture medium and diluted in DMEM (Gibco) enriched with 10% fetal calf serum and antibiotics (penicillin and streptomycin). Cells were harvested with trypsin after 2 h incubation with Colcemid. Afterwards, cell suspension were incubated for 18 minutes in KCl (0,075 M), fixed in 3:1 methanol: acetic acid, then kept at -20°C.

### Classical cytogenetics

Diploid number and chromosome morphology were assessed by the analysis of 20 metaphases with conventional staining (Giemsa 5% in 0.07 M phosphate buffer, pH 6.8). G- and C-banding followed standard protocols [24, 25]. After digital image acquisition, chromosomes were ordered by centromere position and chromosome size.

### Fluorescent *in situ* hybridization

Biotin/Fluorescein labelled 18S/28S and 5S ribosomal DNA probes were used to detect the location of ribosomal RNA gene clusters. Comparative painting was performed using whole chromosome probes generated by flow cytometry at the Cambridge Resource Centre for Comparative Genomics (Cambridge, United Kingdom): chicken, first 10 chromosomes and Z chromosome, and white hawk corresponding to chromosomes homologous to region of GGA1 (LAL 3, 6, 7, 15 and 18), 2 (LAL 2, 4, and 20), 3 (LAL 9, 13, 17 and 26), 4 (LAL 1 and 16), 5 (LAL 5) and 6 (LAL 3) [16]. Hybridization, stringency washes and detection followed standard methodologies previously described [16]. Slides were analyzed using a Zeiss Axioplan2 fluorescent microscope and Axionvisio 4.8 software (Zeiss, Germany).

## Results

### Classical cytogenetics: Conventional staining and banding results

In order to facilitate comparisons with other species, we decided to follow nomenclature taking into account the morphology and size of chromosomes, as proposed by the International System for Standardized Avian Karyotypes [26], instead of the nomenclature based on homology with chicken chromosomes [9–11, 27]. Hence, we present the correspondence of both systems in Table 1.

**Table 1:** Correspondence between nomenclature following chromosome morphology (ours), and homology with GGA [9, 27].

Homology with GGA	Zebra finch [9,27]	Zebra finch, This study (chromosome morphology)	Canary, This study(chromosome morphology)
GGA1p	1	5	5
GGA1q	1A	2	2
GGA2	2	1	1
GGA3	3	3	3
GGA4p	4A	12	12
GGA4q	4	4	4
GGA5	5	6	6
GGA6	6	7	7
GGA7	7	8	8
GGA8	8	9	9
GGA9	9	10	10
GGA10	10	11	11

**Fig 1:**
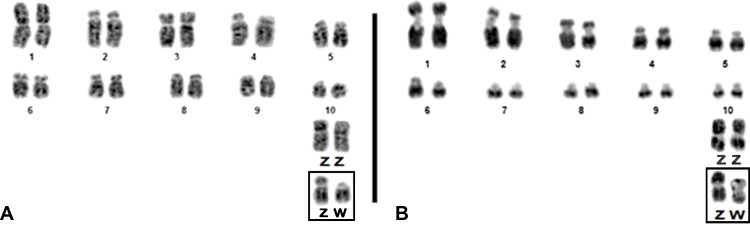
G-banding patterns of the first ten pairs and sex chromosomes of zebra finch (A) and canary (B).

Male and female embryos of both species were studied and their diploid number was found to be 2n = 80. In the zebra finch chromosome 1 is metacentric, chromosomes 3, 4, 5 and Z submetacentric, and chromosomes 2, 6, 7 and 8 acrocentric. All other elements are telocentric. In canary, chromosomes 1–4 are submetacentric, chromosomes 5–10 are acrocentric and the remaining autosomal pairs are telocentric. The Z and the W chromosomes are metacentric. Only the largest chromosome pairs showed some distinguishable G-banding patterns (Fig 1A and 1B).

**Fig 2:**
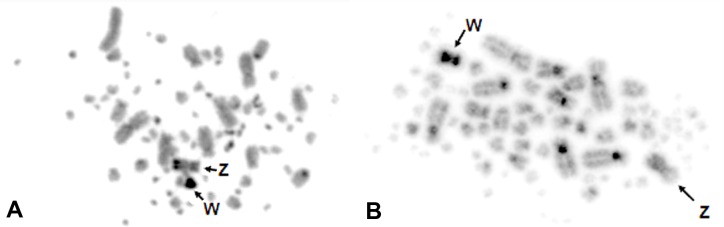
C-band patternsof zebra finch (A) and canary (B). Sex chromosomes are indicated.

In zebra finch, C-banding revealed conspicuous heterochromatic blocks in pairs 6, Z and the W (Fig 2A), while in canary we observed C-positive segments in the centromeric region of all macrochromosomes, in the long arm of chromosome W, and in some microchromosomes (Fig 2B).

### Telomeric sequences and 5S/18S rDNA clusters

**Fig 3:**
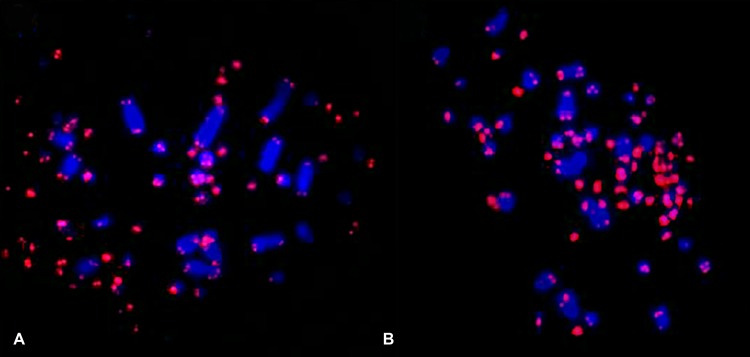
Telomeric probes on chromosomes of zebra finch (A) and canary (B). No interstitial telomeric sequences were observed.

No interstitial telomeric sequences were observed in any of the two species: probes containing telomeric sequences produced signals only in the distal region of chromosome arms, and tended to be brighter in microchromosomes than in macrochromosomes (Fig 3A and 3B).

**Fig 4:**
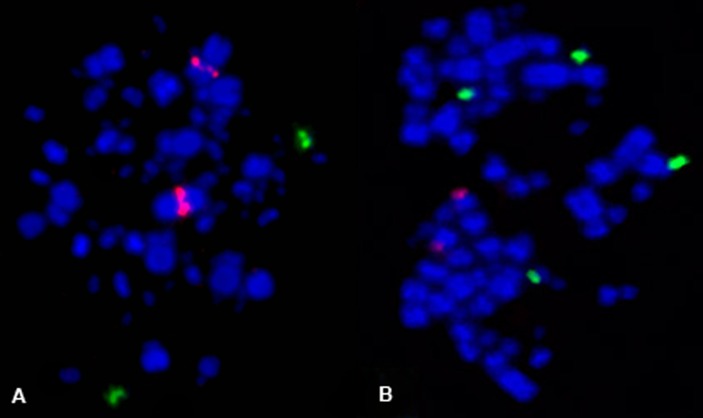
5S (red) and 18/28S (Green) rDNA probes on zebra finch (A) and canary (B).

The rDNA probes revealed some karyotypical differences between the species. 5S rDNA probes hybridized to one cluster in each species. Interestingly, while this cluster was located on a pair of microchromosomes in the canary, in the zebra finch the cluster was located on the medial region of chromosome 2q. Additionally, 18/28S rDNA hybridized onto one pair of microchromosomes of zebra finch, but onto two pairs of microchromosomes in canary (Fig 4A and 4B).

### Chromosome painting

**Fig 5:**
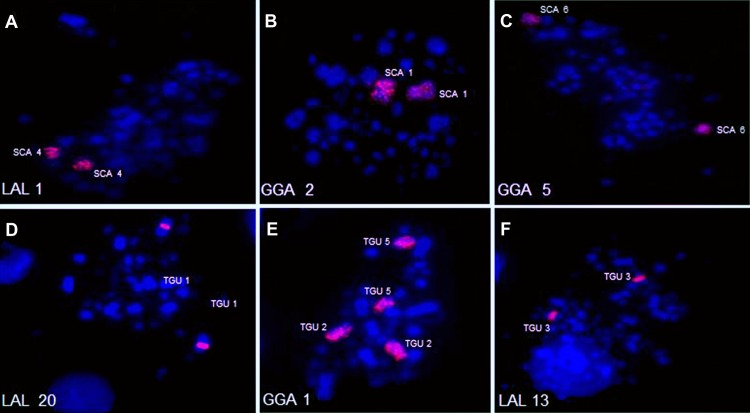
Representative FISH experiments using white hawk (A, D, F) and chicken (B, C, E) probes on metaphase chromosomes of canary (*Serinus canaria*-SCA) [A-C] and zebra finch (*Taeniopygia guttata*-TGU) [D-F].

**Fig 6:**
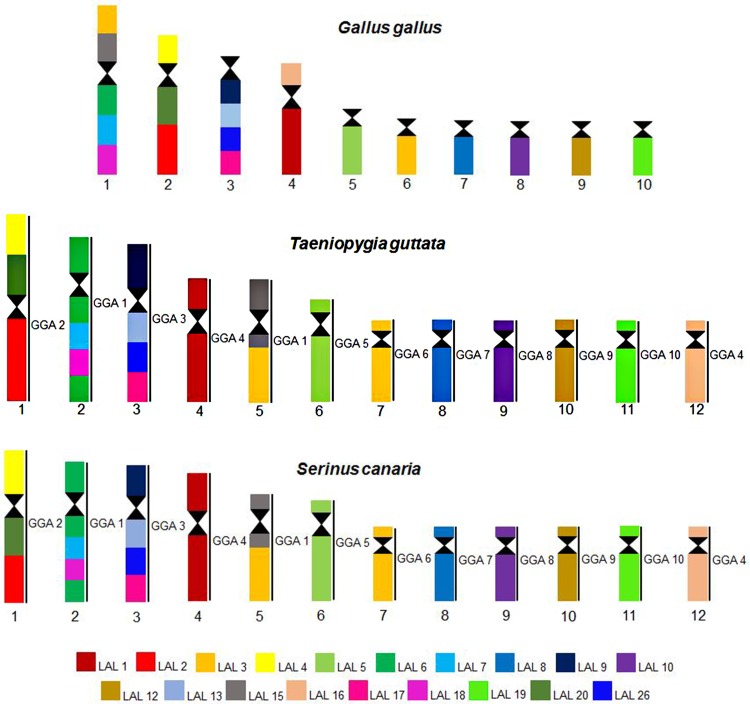
homology maps between chicken (GGA), white hawk (LAL) and two species of Passeriformes (zebra finch and canary).

Examples of experiments using chicken and white hawk probes are shown in Fig 5, while the homology maps of zebra finch and canary with chicken and white hawk are shown in Fig 6.

It can be noticed that the pattern of hybridization using chicken whole chromosome probes were very similar in both species; chromosomes GGA1 and 4 correspond to two different pairs each, and pairs GGA 2, 3, 5, 6, 7, 8, 9 and 10 each correspond to one pair. In canary, a gap was observed in chromosome 2 (which corresponded to GGA1q), corresponding to a block of constitutive heterochromatin.

Experiments using chromosome probes of white hawk revealed that, when compared to chicken syntenic groups, zebra finch and canary exhibited five inversions relative to chicken GGA1: four occurring in the pair corresponding to GGA1q, and one corresponding to GGA1p. In addition, zebra finch presented an inversion in pair 1 (corresponding to GGA2). The occurrence of this inversion explains the morphological differences when we compare TGU1, metacentric, to SCA1, submetacentric. No interchromosomal rearrangement was revealed by any of the probes of chicken applied to zebra finch and canary.

## Discussion

### Karyotype description

The zebra finch and the canary have been used as model species for many different studies. Although both passerine genomes have been fully sequenced and assembled, some aspects of their chromosome organization are still unknown; especially in the canary, published karyotypes rely on conventional staining [11, 17]. The definition of the distribution of constitutive heterochromatic segments and repetitive gene cluster (rDNA) that we report are important for a better understanding and resolution of the *in silico* genome assembly.

The diploid number of 2n = 80 was found both in the zebra-finch and the canary, corroborating previous reports [9–11, 17, 28]. Although the patterns of distribution of the heterochromatic blocks agreed with most reports on avian karyotypes [29], we found in the canary atypical large heterochromatic blocks in some pairs, especially in pair 2, that are not common in macrochromosomes.

### rDNA and telomeric sequences

Although the number and location of repetitive ribosome gene clusters are important features for studies of chromosomal evolution, and cytotaxonomy, these features are still under-investigated in many groups, including birds [30–35]. For instance, in birds, information on the number and distribution of 18/28S is available for a limited number of species, usually using the Ag-NOR technique. In most of these 18/28S rDNA clusters are found on only one pair, usually a microchromosome, although there are already well documented variations [36–39]. For Passeriformes, most species analyzed show only one pair of microchromosomes bearing 18/28S rDNA [22, 40], such as we found in zebra finch and canary. However, there are some species which have 18/28S rDNA clusters on more than one pair [21, 41].

Even more restrictedis data concerning the multigene family 5S rDNA, found only for three species of Galliformes [34, 35], and two songbird species of genus *Saltator* (Passeriformes, Thraupidae) [23]. In all these species, there was only one cluster of 5S rDNA genes, located on one pair of chromosomes, similar in size to pairs 9–11 in Galliformes, and smaller in *Saltator*. The canary shows a similar distribution of 5S rDNA: the probes hybridized only to one pair of microchromosomes. As the identification of the chromosome bearing 5S clusters has been based solely on its size, it cannot be concluded that they are located in the same syntenic groups in both *Saltator* and the canary. In contrast, zebra finch shows a pattern not observed in birds so far; the cluster of 5S rDNA was found in an interstitial position on the long arm of pair 1. As chromosome painting did not detected any interchromosomal rearrangement involving this segment (corresponding to GGA1q) transposition is a possible explanation [42]. It seems that the comparative analysis of these clusters can give valuable information about karyotypical diversity during genome evolution of birds, as observed in other groups of vertebrates [43, 44]. Unfortunately, our attempt to match our findings with expectations based on genome sequences did not succeed. The blast of ribosomal genes from chicken to chicken genomes indicated that there is no clear annotation and/or overlap of the rRNAs chicken genes even using the best annotated genome and complete sequences of rRNA genes published. The most plausible reason being the excessive enrichment of repetitive sequences and artifacts of the assembly procedures of the genomes. As discussed by Dyomin et al. [45] there is no complete annotation of rRNA genes in avian genomes so far despite all efforts made.

Although many species of birds have interstitial telomeric sequences, including Passeriformes such as the Redwing (*Turdus iliacus*) and the chaffinch (*Fringilla coelebs*) [19], in canary and zebra finch telomeric sequences were restricted to the terminal regions of chromosomes. In addition, in accordance with other studies, we observed more intense telomeric probe signals in microchromosomes [19, 46–49]. According to Nanda et al. [48], the high density of (TTAGGG)_n_ repeats in microchromosomes may contribute to the high meiotic recombination rate observed in these elements. In addition, previous studies have shown a higher number of interstitial telomeric sequences in Ratitas and Galloanserae, which led us to propose that this may represent a plesiomorphic condition, tending to diminish during avian evolution, despite the conservation of diploid number and syntenic groups.

### Chromosome painting using chicken and white hawk probes

In general, zebra finch and canary show the conservation of syntenic groups. When compared to the putative ancestral karyotype, the only difference found is the fission of the first chromosome pair into two distinct elements. Furthermore, white-hawk probes confirmed most of the inversions found in other species of Passeriformes, reinforcing that these inversions, as well as the fission of pair 1, occurred very early in the history of this order.

Each chicken probe hybridized to one pair of chromosome of zebra finch and canary, except for probes GGA1 and GGA4, which hybridized to two pairs each. As GGA4 corresponds to two elements universally in different groups of birds, confirming it as the ancestral state [16], the centric fission of GGA1 seems to be apomorphic for Passeriformes. This has been described in all the species analyzed by FISH so far [18–23]. Likewise, the use of white hawk probes confirmed the results found in other species of Passeriformes, both Oscines [21, 23] and Suboscines [22], revealing the occurrence of a group of sequential chromosomal inversions in segments homologous to GGA1. However, LAL 18, which corresponds to one block in the species analyzed here, and also in *Saltator*, is homologous to two different segments in *Turdus* and *Elaenia*, that belong to Oscines and Suboscines, respectively [21, 23]. These findings may be explained by two different alternative scenarios. First, and maybe more parsimonious, it could be that this inversion, in which the segment homologous to LAL 18 was split into two parts, occurred some time before the split of Oscines and Suboscines. On the other hand, an alternative hypothesis based on the high level of recurrent breakpoints in birds [3] could consider a 4th inversion in *Saltator* species, in the canary and the zebra finch, which would have reverted the segment homologous to LAL 18 to one continuous block [23]. This proposal is supported by the fact that the genera *Saltator*, *Serinus* and *Taeniopygia* are included in the same phylogenetic branch, called “Core Passeroidea” [50]. The 4th inversion would have occurred in the common ancestor of this group. In any case, we are aware that only the analyses of other genera belonging to these groups may confirm one of these hypotheses.

**Fig 7:**
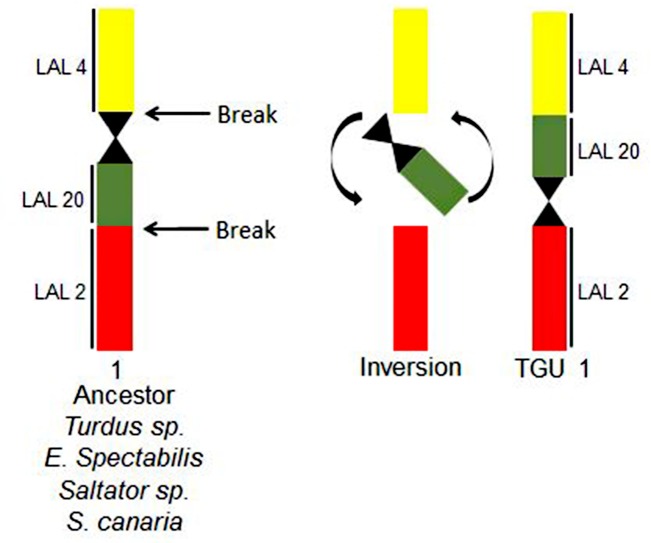
Schematic representation of the inversion found in TGU1 (GGA2).

Concerning the comparison of rearrangements found in the canary and zebra finch, it is important to consider that the inversions detected by the use of white hawk probes in pairs 1 (GGA2), 2 (GGA1q) and 5 (GGA1p) corroborate the proposals of Warren et al. [8] in zebra finch, and by Frankl et al. [11] in canary, using the genomic *in silico* assembly data. Considering other species with data on genome sequence alignment, the occurrence of these inversions can be expanded to four other species of this group: *Geospiza fortis*, *Zonotrichia albicollis*, *Pseudopodoces humilis* and *Ficedula albicollis* [11]. In addition, the comparison of our data to previous studies indicated that TGU1 had one additional pericentric inversion when compared to *Turdus*, *Elaenia* and *Saltador* [21, 23] (Fig 7). Hence, while in these genera and in canary the first pair is submetacentric, in zebra finch it is metacentric. This inversion was detected by Warren et al. [8] by sequence alignment. It was proposed that some of the inversions described by Skinner & Griffin [3] in the genome study of zebra finch must be autapomorphies of this species, and not shared by others so far [11].

## Conclusions

The genome sequencing of an increasing number of birds, combined with cytogenomic mapping is helping to diminish the discrepancy of information concerning avian genomic organization in comparison to other Vertebrate groups. Additionally, the agreement between the data obtained by *in silico* assembling and FISH approaches show that these metholologies are complementary and may be used in combination to generate cytogenetic markers, or to provide information not easily obtained by sequencing and genomic assembling, such as the localization of repetitive sequences, as for example 5S and 18S rDNA clusters. Hence, a complete karyotypical characterization–including the distribution of heterochromatic blocks and rDNA blocks–may be important to complete the interpretation of data obtained by sequencing. A clear example of this is the fact that, because of the peculiar nature of the avian karyotype, the number of syntenic groups generated by bioinformatic approaches are not accurate, and usually do not match with the actual diploid number [11].
